# Flt3L Combined with Rapamycin Promotes Cardiac Allograft Tolerance by Inducing Regulatory Dendritic Cells and Allograft Autophagy in Mice

**DOI:** 10.1371/journal.pone.0046230

**Published:** 2012-10-02

**Authors:** Ali Xiong, Lihua Duan, Jie Chen, Zhigang Fan, Fang Zheng, Zheng Tan, Feili Gong, Min Fang

**Affiliations:** 1 Department of Immunology, Tongji Medical College, Huazhong University of Science and Technology, Wuhan, Hubei, China; 2 Key Laboratory of Organ Transplantation, Ministry of Education, Wuhan, Hubei, China; 3 Key Laboratory of Organ Transplantation, Ministry of Health, Wuhan, Hubei, China; University of Michigan School of Medicine, United States of America

## Abstract

The induction of immune tolerance is still a formidable challenge in organ transplantation. Dendritic cells (DCs) play an important role in orchestrating immune responses by either mediating protective immune responses or inducing antigen specific tolerance. Previous studies demonstrated that the fms-like tyrosine kinase 3 receptor (Flt3) and its ligand (Flt3L) play an essential role in the regulation of DC commitment and development. Here, we report a synergic effect between Flt3L and low-dose rapamycin (Rapa) in the protection of allograft rejction. It was found that Flt3L combined with Rapa significantly prolonged murine cardiac allograft survival time as compared with that of untreated recipients or recipients treated with Rapa or Flt3L alone. Mechanistic studies revealed that Flt3L combined with low-dose of Rapa induced the generation of tolerogenic DCs along with the production of CD25^+^ Foxp3^+^ regulatory T cells and IL-10 secretion. We also observed enhanced autophagy in the cardiac allograft, which could be another asset contributing to the enhanced allograft survival. All together, these data suggest that Flt3L combined with low-dose of Rapa could be an effective therapeutic approach to induce tolerance in clinical setting of transplantation.

## Introduction

Organ transplantation has become the primary treatment for patients with end-stage organ failure [1]. Although the application of immunosuppressive drugs has contributed significantly to the success of allograft survival, side effects resulted from immunosuppression or drug toxicity also markedly impact the quality of life of recipients [2], [3]. Therefore, establishment of strategies aimed at inducing allograft tolerance is wanting.

In the setting of transplantation, DCs actively mediate graft rejection by presenting donor-derived alloantigens to naïve T cells. However, there is emerging evidence indicating that other than mediation of allograft rejection, DCs also possess the capability to induce allograft tolerance [4]. Therefore, DCs are capable of inducing either immune response or tolerance depending on their activation and maturation status [5], [6].

Flt3, a member of the tyrosine-kinase receptor family, was initially cloned from fetal liver of cells with hematopoietic stem cell activity [7]. Flt3L is the ligand for Flt3, which is a key regulator for DC commitment and development. Mice administered with Flt3L displayed the expansion of certain subtypes of DCs such as the plasmacytoid DCs (pDCs) and conventional DCs (cDCs) in the spleen [8]. Studies have shown that pDCs play an important role in the induction of Tregs in vivo manifested by that their precursors are able to prolong graft survival [9]. Studies in hematopoietic cell transplantation also revealed that Flt3L is a potent mobilizer to induce murine hematopoietic chimerism, while long-term persistence of donor hematopoietic cells in peripheral lymphoid and non-lymphoid tissues of organ allograft recipients is postulated to be an essential prerequisite for the induction of donor-specific tolerance [10], [11].

Autophagy in eukaryotic cells is a cellular quality control process to deliver cytoplasmic constituents for lysosomal degradation, which enables cells to recycle nutrients for survival during nutrient starvation [12]. Rapamycin, an inhibitor of the mammalian target of rapamycin (mTOR) has been used to stimulate autophagy. In the steady state, autophagy-mediated antigen processing in thymic epithelial cells could have a crucial role in the induction and maintenance of CD4^+^ T-cell tolerance [12], [13].

In this study, we examined the effects of Flt3L in combination with Rapamycin in a BALB/c-to-C57BL/6 cardiac allograft model. Our results demonstrate that Flt3L combined with low-dose of Rapamycin prevented acute allograft rejection and prolonged allograft survival time. The enhanced allograft survival is associated with the induction of tolerogenic DCs along with the production of CD25^+^ Foxp3^+^ regulatory T cells and increased allograft autophagy. Our data suggest that Flt3L combined with low-dose of Rapa could be a promising therapeutic strategy in clinical transplantation.

## Results

### Flt3L Combined with Low-dose of Rapa Prolongs Cardiac Allograft Survival

**Figure 1:**
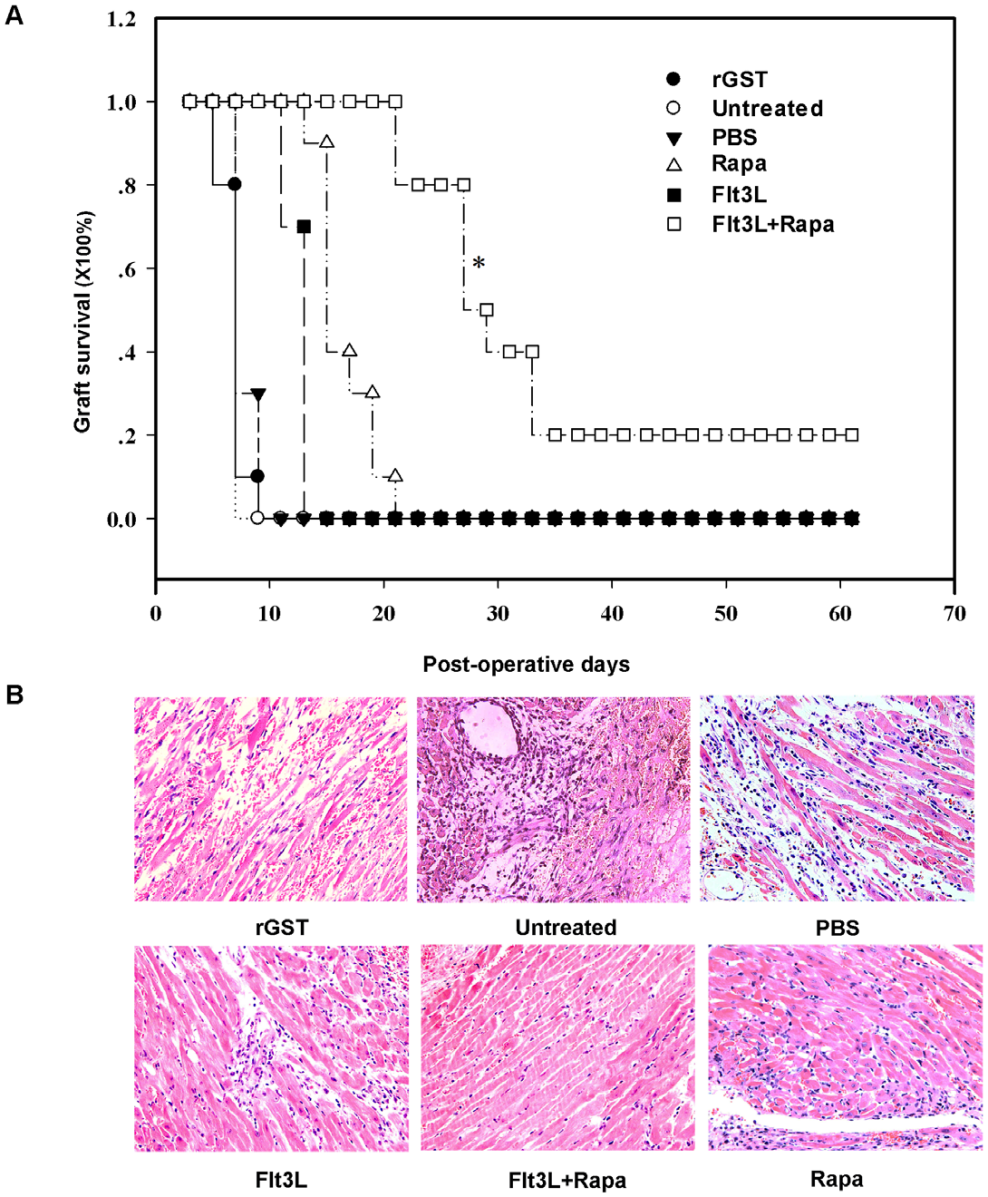
Flt3L combined with low-dose of Rapa prolongs cardiac allograft survival and attenuates infiltrates in the graft. (A) Heart allograft survival. The cardiac allograft survival time in recipients treated with Flt3L (10 µg/day, i.v.) and low-dose of Rapa (2 mg/kg/day; p.o, POD0-15) was significant longer than those of the untreated and monotherapy groups (n = 10) (* *P*<0.05, combination therapy group vs. other groups). (B) Histology of cardiac allografts. Grafts were harvested at the time of rejection or at study endpoint (POD 100) and evaluated by H&E staining of paraffin sections (original magnification, ×200). The results are representative of three independently performed experiments.

We first sought to investigate the regulatory effect of Flt3L combined with a short-term of low-dose Rapa treatment on allograft acute rejection. A murine cardiac allograft transplantation model was employed to address this question. As shown in Figure 1A, untreated recipients rapidly rejected their grafts (7.6±0.516 days) along with typical pathological features of acute rejection (Fig. 1B). Similar pathological features were observed in recipients treated with either PBS or rGST (controls, Figure 1B). As expected, administration of Rapa significantly increased graft mean survival time (MST) (16.9±2.767 days, Fig. 1A). Similarly, recipients treated with Flt3L doubled allograft MST (13.1±1.287 days, Fig. 1A). In sharp contrast, a significant enhanced graft survival was observed in mice treated with Flt3L and low-dose of Rapa (36.4±18.704 days, Fig. 1A). Remarkably, about 20% of recipients showed long-term graft survival (MST>100 days, Fig. 1A). In consistent with these results, a significant reduction for inflammatory infiltration was observed in the allograft sections (Fig. 1B).

### Flt3L Combined with Rapa Induces Tolerogenic DCs in the Recipient Mice

**Figure 2:**
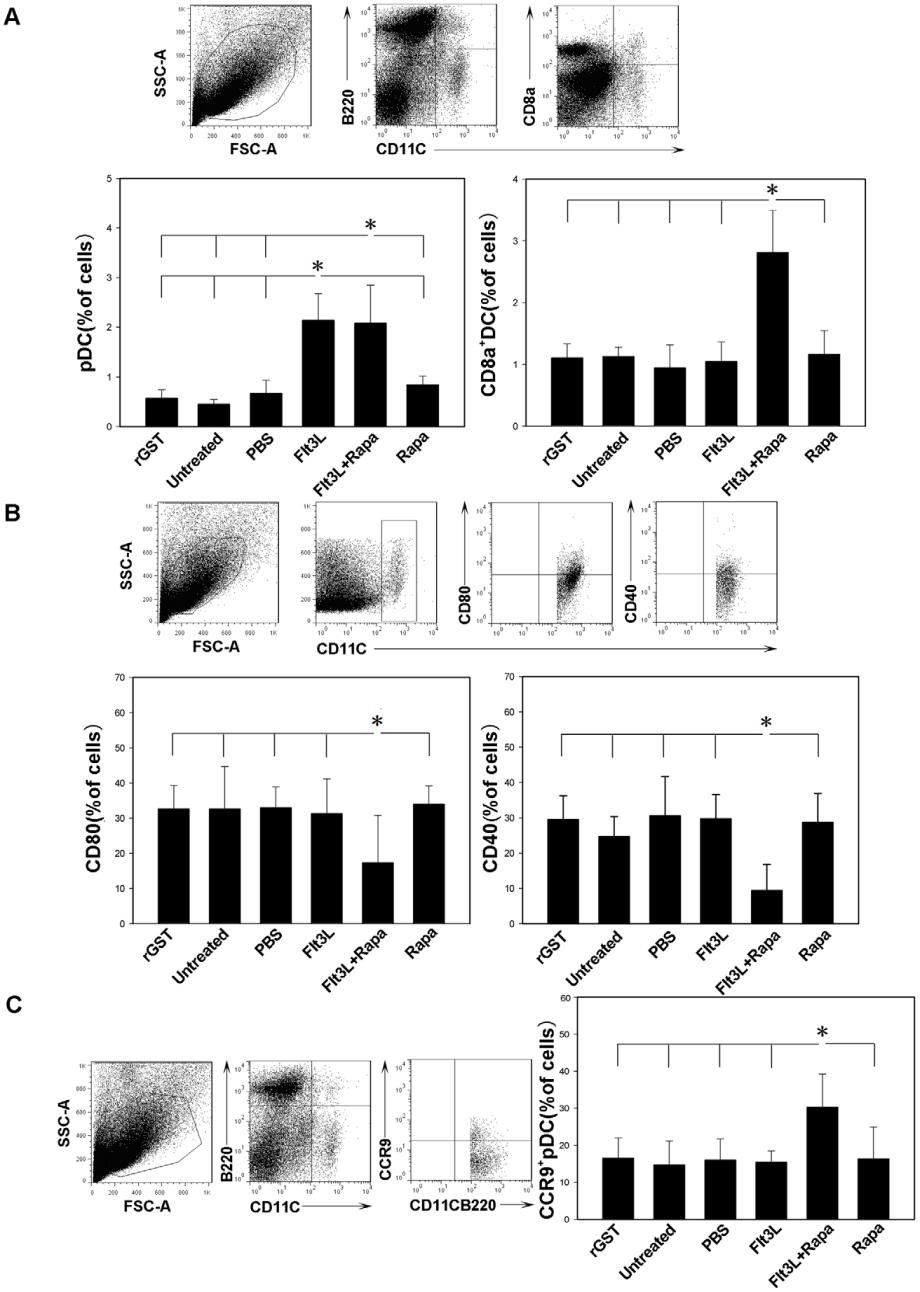
DCs from recipients treated with Flt3L/Rapa display a tolerogenic phenotype. Spleen cells isolated from mice treated with Flt3L/Rapa and other control mice at the time of rejection or at study endpoint (POD 100) were stained with PE-cy7-labeled anti-mouse CD11c mAb, Per-cy5.5-labeled anti-mouse B220 mAb, APC-cy7-labeled anti-mouse CD8a mAb, FITC-labeled anti-mouse CCR9 mAb, APC-labeled anti-mouse CD80 mAb and APC-labeled anti-mouse CD40 mAb. CD11c^+^ and CD11c^+^ B220^+^ gated DCs were analyzed by flow cytometry for expression of the various DC markers. (A) The bar graph was a summary of percentages of pDC and CD8a^+^ DC in the recipients. (B) CD11c^+^-gated DCs were analyzed for expression of costimulatory markers CD80 and CD40 to assess their maturation state. (C) The percentage of CCR9^+^ cell of gated pDC subsets was assessed by FACS analysis. The results are representative of three independently performed experiments (* P<0.05).

We next examined DC phenotypes in the recipient mice. It was noted that a significant increase in the number of pDCs in mice treated with Flt3L or Flt3L with low-dose of Rapa as compared with that of control mice (Fig. 2A). Similarly, we also detected a significantly higher proportion of splenic CD8a^+^ DCs in Flt3L or Flt3L/Rapa treated mice (Fig. 2A). Since both CD8a^+^ DCs and pDCs have regulatory functions [14], Our data suggest that Flt3Lcombined with Rapa could induce the expansion of regulatory DCs in the recipient mice.

Rapamycin has been shown to inhibit the up-regulation of maturation markers expressed by DCs [15], while immature DCs have been demonstrated with capability to prolong allograft survival [16], [17]. In line with this notion, Flt3L combined with Rapa significantly blocked CD11c^+^ DC maturation as manifested by the decreased CD40, CD80 expression (Fig. 2B). Since CCR9^+^ pDCs had previously been reported to be potent inducers for Tregs [18], we then investigated whether CCR9^+^ pDCs are implicated in the induction of allograft tolerance. Indeed, recipients treated with Flt3L and low-dose of Rapa displayed a significant increase for CCR9^+^ pDCs as compared with those recipients treated with Flt3L or Rapa alone or other groups (Fig. 2C).

### Flt3L Combined with Rapa Promotes the Production of CD4^+^ CD25^+^ Foxp3^+^ and CD8^+^ CD25^+^ Foxp3^+^ T Cells

**Figure 3:**
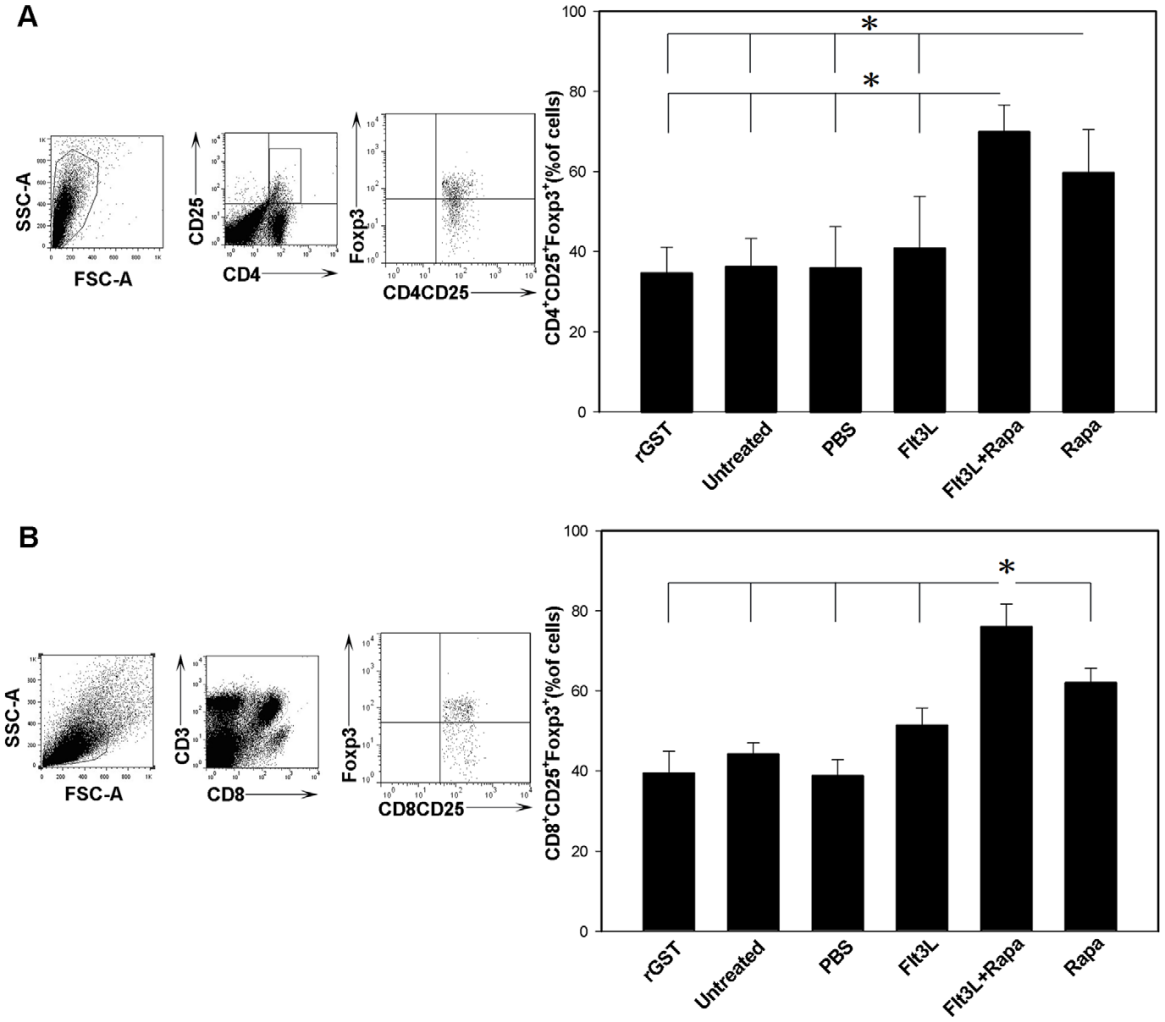
Flt3L combined with Rapa promotes the production of CD4^+^CD25^+^Foxp3^+^ and CD8^+^CD25^+^Foxp3^+^ T cells. Spleen cells isolated from mice treated with Flt3L/Rapa and other control mice at the time of rejection or at study endpoint (POD 100) were analyzed to determine the proportion of CD4^+^ CD25^+^ Foxp3^+^ and CD8^+^ CD25^+^ Foxp3^+^ T cells. (A, B) The expression of Foxp3 by CD4^+^ CD25^+^ and CD8^+^ CD25^+^ T cells was analyzed by flow cytometry after intracellular staining. The bar graph was a summary of percentages of CD4^+^ CD25^+^ Foxp3^+^ T cells and CD8^+^ CD25^+^ Foxp3^+^ T cells in the recipients. The data shown are representative of three independent experiments that yielded comparable results (* *P*<0.05).

The above results prompted us to examine the proportion of CD4^+^CD25^+^Foxp3^+^ and CD8^+^CD25^+^Foxp3^+^ Tregs. To this end, splenic T cells isolated from each group of recipient mice were stained for intracellular Foxp3 after co-staining with anti-CD4/CD25 antibodies or anti-CD3/CD8/CD25 antibodies, and the cells were next analyzed by flow cytometry. A significant increase of CD4^+^ CD25^+^ Foxp3^+^ and CD8^+^CD25^+^Foxp3^+^ Tregs was observed in recipients treated with Flt3L and Rapa as compared with recipients in other groups (Fig. 3).

### Flt3L/Rapa Therapy Favors Anergic Induction in Alloreactive T Cells

**Figure 4:**
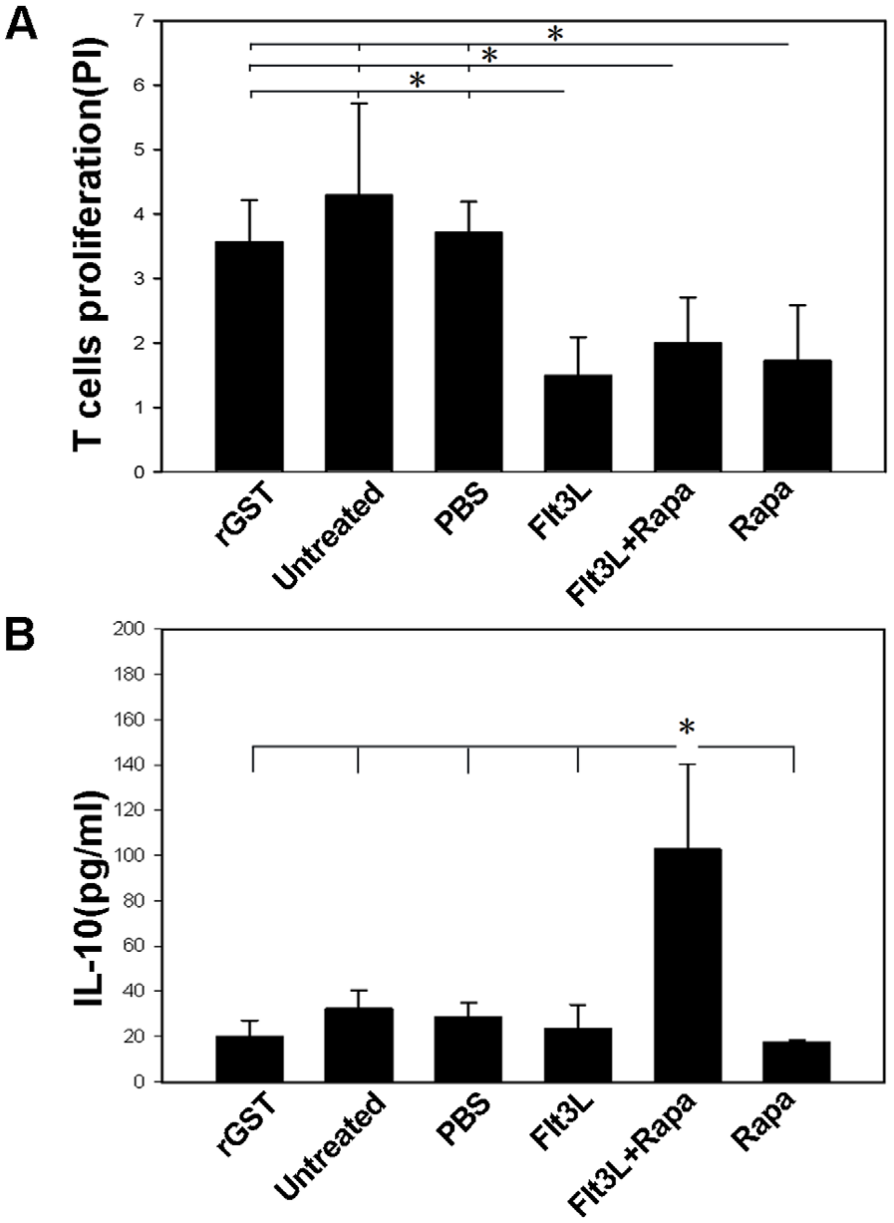
Flt3L/Rapa therapy favors anergic induction in alloreactive T cells. (A) Splenic T cells originated from cardiac allograft recipient mice at the time of rejection or at study endpoint (POD 100) were used as responder cells for MLR assay with donor-derived DCs (DC: T ratio of 1∶5). Bar graph was the summary of proliferation index (PI) of T cells isolated from different recipients. (B) The culture supernatants were harvested and cytokine IL-10 levels were quantification by ELISA. The data shown are representative of three independent experiments (*P<0.05).

We then tested the reactivity of bulk T cells isolated from the spleens of the recipients against donor-derived DCs. As shown in Figure 4A, T cells derived from Flt3L/Rapa treated mice exhibited a significantly lower proliferative response against donor BALB/c DCs in MLR when compared with that of T cells derived from untreated, rGST-treated and PBS-treated recipients. Unexpectedly, a similar proliferation rate was noted in recipients treated with either Flt3L or Rapa alone as compared with that of recipients treated with Flt3L/Rapa. This result promoted us to further examine cytokine production in the culture supernatants by ELISA. Of Interestingly note, the production of IL-10 was significantly higher in the Flt3L/Rapa treated mice. More importantly, although T cells derived from Flt3L or Rapa treated mice showed similar potency for proliferation (Fig. 4A), but their capacity for secretion of IL-10 was similar to those T cells from control mice (Fig. 4B). Collectively, our data suggest that Flt3L/Rapa treatment enhances IL-10 secretion which could favor the induction of T anergy.

### CD8^+^ T Cells and pDCs Play a Dominant Role to Promote Allograft Long-term Survival

**Figure 5:**
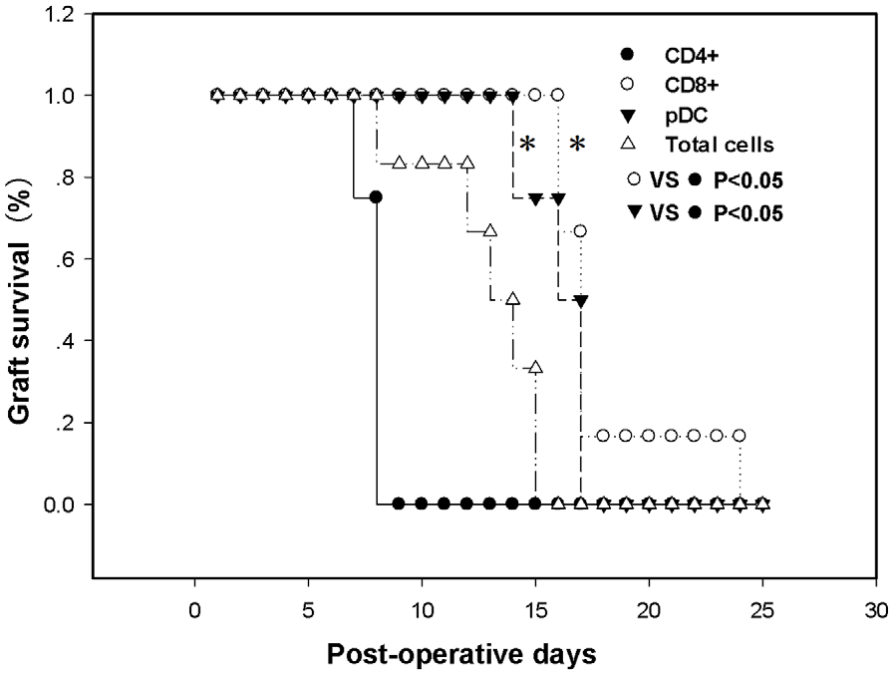
CD8^+^ T cells and pDCs play a dominant role to promote allograft long-term survival. The purified CD4^+^ T cells (5×10^6^), CD8^+^ T cells (5×10^6^), pDCs (5×10^5^) and total splenotytes (5×10^6^) originated from recipients with long-term allograft survival were then adoptively transferred into naive recipients respectively. One day after the adoptive transfer, the mice were transplanted with cardiac allograft. The survival time of cardiac allografts was monitored. The data shown are representative of three independent experiments (*P<0.05).

Given that Flt3L/Rapa therapy resulted in reproducible long-term allograft survival in about 20% of recipients without immunosuppression (Fig. 1A), we thus employed adoptive transfer studies to characterize the cell subpopulations for sustained allograft survival. For this purpose, CD4^+^ T cells, CD8^+^ T cells, pDCs and total splenocytes were first isolated from long-term survival recipients, and then adoptively transferred into naive recipients. The mice were next transplanted with cardiac allografts as described. As shown in Figure 5, transferred CD8^+^ T cells and pDCs exhibited the ability to prolong allograft survival. Unexpectedly, compared with CD8^+^ T cells and pDCs, adoptive transfer of CD4^+^ T cells failed to provide protection against allograft rejection.

### Flt3L/Rapa Therapy Enhances Autophagy in the Grafts

**Figure 6:**
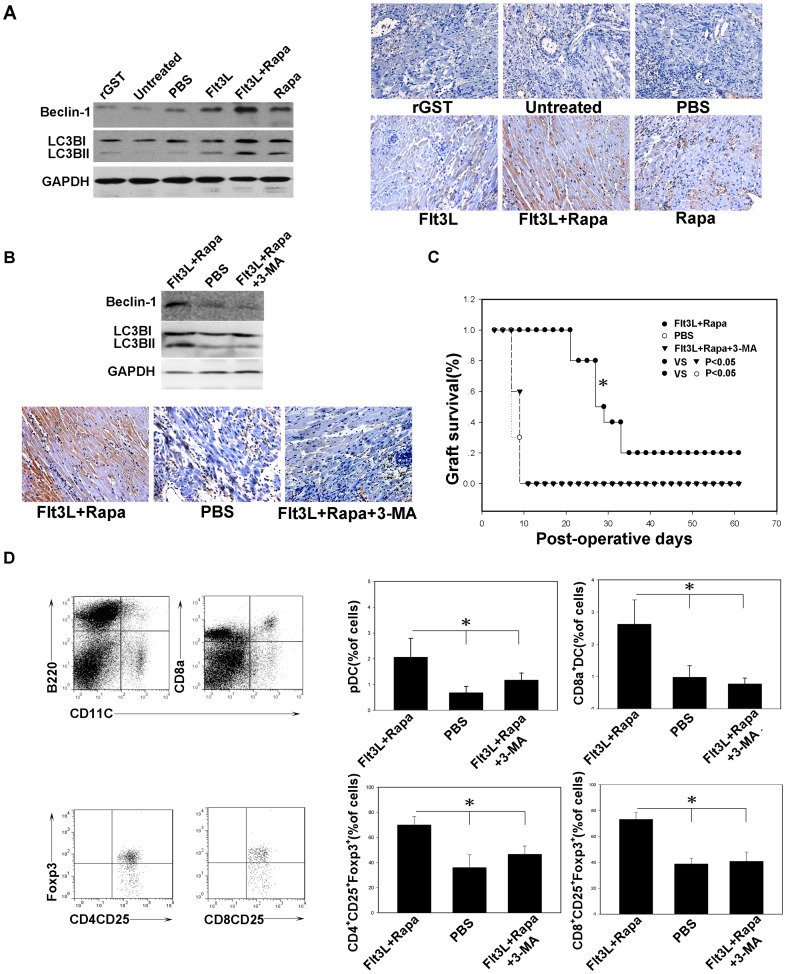
Treatment with Flt3L/Rapa enhances the autophagy in the cardiac allograft. (A, B) The cytoplasmic extracts of cardiac allograft tissue were subjected to Western blotting for evaluating the expression level of Beclin-1 and LC3B, Immunohistochemistry was conducted for examining the expression of Beclin-1 by the cardiac allograft of each group (original magnification, ×200). (C) Heart allograft survival. The cardiac allograft survival time in recipients treated with Flt3L/Rapa and 3-MA (24 mg/kg, i.p, 5, 10, 15 days) was significant shorter than that of the recipients treated with Flt3L/Rapa (* *P*<0.05). (D) The percentage of pDC, CD8a^+^ DC, CD4^+^ CD25^+^ Foxp3^+^ and CD8^+^ CD25^+^ Foxp3^+^ T cells was assessed by FACS analysis (* *P*<0.05, combination therapy group vs. other groups at endpoint). All data presented here are representative of three separate experiments with consistent results.

To determine whether the prolonged allograft survival was associated with enhanced autophagy, we examined the levels of autophagy-related protein beclin-1, microtubule associated protein1 light chain 3 (LC3) I and II by Western blot analysis. As shown in Fig. 6A, only low levels of Beclin-1 and LC3 II were detected in control mice (rGST, untreated and PBS groups), but higher levels of Beclin-1 and LC3 II were observed in mice treated with Flt3L or Rapa alone. Importantly, the highest Beclin-1 and LC3 II expressions were noted in Flt3L/Rapa treated mice. To further confirm these results, we did immunohistochemical analysis, and similar results were obtained (Fig. 6A). To examine the functional relevance for the enhanced autophagy in allograft survival, we performed similar transplantation along with the administration of 3-MA, an autophagy inhibitor [19], [20]. As expected, administration of 3-MA significantly repressed autophagy as determined by Western blot and immunohitochemical analysis (Fig. 6B). Remarkably, repression of autophagy significantly attenuated Flt3L/Rapa therapy mediated protection against allograft rejection (Fig. 6C). In consistent with this result, we also observed a decrease for the number of regulatory T cells and regulatory DCs (Fig. 6D). Taken together, these data indicate that autophagy plays an essential role in Flt3L/Rapa therapy mediated protection against allograft rejection.

## Discussion

Despite the benefit of Flt3L and Rapa for anti-rejection in setting of transplantation, the effect for Flt3L or Rapa based mono-therapy on allograft survival remains poor [21], [22], [23], [24]. In the present study, we have demonstrated that Flt3Lcombined with low-dose of Rapa could markedly increase graft survival time as compared with that of Flt3L or Rapa alone. The enhanced protective effect was achieved by promoting the generation of tolerogenic DCs and Tregs along with increased autophagy in the allograft.

It has been reported that DCs expanded by Flt3L only express low levels of costimulatory molecules, and therefore, they are able to delay allograft rejection in transplantation settings [25], [26]. In contrast to Flt3L or Rapa alone, our data show that Flt3L combined with low-dose of Rapa are more potent to expand DCs with tolerogenic characteristics. Moreover, short-term of low-dose Rapa exerts the effect to expand Tregs while to avoid side effect due to high-dose of Rapa.

Several DC subsets have been reported to promote tolerance induction [4], [27]. The CD11c^+^ CD8a^+^ DC subset could promote peripheral self-tolerance and prolong the survival of cardiac allografts in rodents [28], [29]. More recently, pDC precursors have been identified and are implicated in the induction of allogeneic T-cell hyporesponsiveness [9], [30]. In line with these findings, our phenotypic analysis of splenic DCs in the recipient mice revealed that Flt3L/Rapa treatment increased the proportion of DC subsets with regulatory effect such as CD11c^+^ CD8a^+^ DCs and pDCs. It is known that immature DCs not only fail to prime T cells effectively, but also serve to promote tolerance induction [31]. Our studies show that Flt3L combined with Rapa could block DC maturation and increase the ratio of CCR9^+^ pDCs, while CCR9^+^ pDCs are immature pDCs with potency to suppress antigen-specific immune responses both in vitro and in vivo [18].

Regulatory T cells are a subpopulation of T cells that have the ability to suppress immune reactions and maintain tolerance. They were believed to play a key role in transplant tolerance induction. The most important representative of regulatory T cells is the naturally occurring regulatory T cells (Tregs), with a CD4^+^ CD25^+^ Foxp3^+^ phenotype. They were found to mediate donor-specific unresponsiveness in some stable renal [32], [33], [34], [35], [36] and liver [37] transplant patients. In addition, adoptive transfers of Tregs could prevent allograft rejection and prolong graft survival in the mouse models [38]. Previously, addition of rapamycin to human and murine T cell cultures preferentially expanded regulatory T cells [39], [40]. Our data indicated that infusion of Flt3L with Rapa and Rapa alone enhances the production of CD4^+^ CD25^+^ Foxp3^+^ Tregs. In addition, we found a significant increase of splenic CD8^+^CD25^+^Foxp3^+^ T cell populations in the recipient mice treated with Flt3L/Rapa, while CD8^+^CD25^+^Foxp3^+^ T cell have been found being able to inhibit antigen-specific CD4^+^ and CD8^+^ T cell responses by cell contact inhibition, secretion of cytokines and other ways [41].

Our adoptive transfer studies showed that CD8^+^ T cells are responsible for induction of long-lasting tolerance toward donor alloantigen. We therefore proposed that Flt3L-mobilized recipient DCs might process indirect antigen presentation of allopeptides to induce tolerance for host CD8^+^ alloreactive T cells, which are the main effector mechanism of cellular rejection across an MHC class I barrier. This notion is well supported by a recent study, in which Rapa-conditioned, alloAg-pulsed DC can present acquired MHC-peptide complexes from donor cells and modulate directly-reactive CD8^+^ T cell function, and through which, these DCs enhance Ag-specific CD8^+^ T cell undergoing apoptosis [42]. Furthermore, CD8^+^CD28^–^T cells may also play an important role in tolerance induction. These T cells represent a transient state of effector CD8^+^ T cells, which promotes the production of the immunosuppressive cytokine IL-10 [43], [44]. Emerging data suggest that the appearance of these T cells in transplant recipients is associated with better graft acceptance along with stable function [45], [46], [47].

Finally, our data indicate that autophagy plays an important role in the protective effect of Flt3L in combination with Rapa on cardiac allograft survival. The possible mechanisms is that autophagy delivers self-proteins for MHC class II loading, and their peptidic fragments are essential for the deletion of self-reactive T cells in the thymus. Recent studies have also suggested that the possible link between autophagy and tolerance is that autophagy plays a role in the removal of apoptotic cell corpses [48]. Regarding to the effect of autophagy on allograft survival seems controversial. In a rat liver transplantation study, it has been shown that autophagy-associated hepatocyte death triggers liver graft dysfunction, and suppression of autophagy prevent cold ischemia-warm reperfusion injury associated with liver transplantation [49]. Thus, further relevant investigations are needed.

### Conclusion

In summary, Flt3L combined with Rapamycin is able to promote the prolongation of allograft survival. This protective effect is associated with the generation of tolerogenic DC subsets, CD25^+^ Foxp3^+^ regulatory T cells (Tregs), and enhanced allograft autophagy.

## Materials and Methods

### Mice

Six to eight week-old female C57BL/6 (H-2^b^), BALB/c (H-2^d^) mice were obtained from the animal facilities at Tongji Medical College. All mice were maintained under specific pathogen-free conditions and the studies were carried out in compliance with the institutional animal care and use guidelines.

### Reagents and Antibodies

CFSE was purchased from Invitrogen (Eugene, OR, USA). All kits were obtained as follows: Mouse IL-10 ELISA MAX™ Deluxe was derived from Biolegend. Mouse CD4 (L3T4) MicroBeads (Miltenyi Biotec, CA), Mouse Plasmacytoid Dendritic Cell Isolation Kit II (Miltenyi Biotec, CA), Mouse CD8a (Ly-2) MicroBeads were derived from Miltenyi Biotec, CA. Rapamycin was purchased from Wyeth, USA. 3-Methyladenine was purchased from Sigma. Antibodies for Beclin-1,and LC3B were purchased from cell signaling. PE-cy7-labeled anti-Mouse CD11c (clone N418), Per-cy5.5-labeled anti-Mouse B220 (clone RA3-6B2), FITC-labeled anti-Mouse CCR9 (clone CW-1.2), PE-labeled anti-rat CD3 (clone G4.18), PE-cy7-labeled anti-Mouse CD3 (clone 145-2C11), APC-labeled anti-Mouse CD80 (clone 16-10A1), APC-labeled anti-Mouse CD40 (clone 1C10), anti-Mouse CD16/32 (clone 93), FITC-labeled anti-Mouse CD4 (clone GK1.5), PE-labeled anti-Mouse CD25 (clone PC61.5), APC-cy7-labeled anti-Mouse CD8a (clone 53-6.7), APC-labeled anti-Mouse/Rat Foxp3 (clone FJK-16s) were obtained from eBioscience (San Diego, CA, USA).

### Expression and Purification of Recombinant Flt3L

DNA sequence encoding Flt3L (696 bp) was amplified from the C57BL/6 mouse spleen cDNA using the following oligonucleotides: 5′-CGG GAT CCA TGA CAG TGC TGG CGC C-3′ and 5′-CGG AAT TCA TCC TAG GGA TGG GAG-3′. The resulting products were subsequently cloned into a pGEX-4T3 vector with a glutathione S-transferase tag. The plasmid was transformed into Escherichia coli strain BL21. Recombinant Flt3L expression was induced by addition of 1 mM IPTG and then purified using the glutathione sepharose 4B resin columns as instructed by the manufacturer. A GST vector protein was also expressed and purified to homogeneity. The proteins were passed over polymyxin B columns (PIERCE) to remove any contaminated LPS and further concentrated by 3KD Micropore Filters. The purified recombinant Flt3L and rGST were stored in aliquots at −80°C until use.

### Heart Transplantation Model

Heterotopic cardiac transplantation was performed as previously described [50]. In this study, transplant surgery involved the transfer of fully MHC-mismatched hearts from BALB/c (H-2^d^) donors to C57BL/6 (H-2^b^) recipients. Heart beat of the grafts was monitored and evaluated daily by direct abdominal palpation to detect the state of cardiac health/rejection.

### Experimental Groups

Heart transplant recipients were randomly assigned to six groups (n = 10): Group 1, rGST alone; Group 2, untreated control; Group 3, PBS alone; Group 4, Flt3L alone (10 µg/day, i.v) for 10 consecutive days before heart transplantation; Group 5, Rapa alone (2 mg/kg/day; p.o, POD0-15); Group 6, combination treatment of Flt3L and Rapa.

### Graft Histology

Allograft samples were fixed in 4% paraformaldehyde. Serial sections (5 µm in thickness) were prepared using a microtome and stained with hematoxylin/eosin for the analysis of pathological changes.

### Flow Cytometric Analysis

Spleen cells isolated from mice treated with Flt3L/Rapa and other control mice were incubated with anti-CD16/CD32 monoclonal antibody to block FcR, and then stained with PE-cy7-labeled anti-mouse CD11c mAb, Per-cy5.5-labeled anti-mouse B220 mAb, APC-cy7-labeled anti-CD8a mAb, FITC-labeled anti-mouse CCR9 mAb, APC-labeled anti-mouse CD80 mAb and APC-labeled anti-mouse CD40 mAb. CD11c^+^ and CD11c^+^ B220^+^ gated DCs were analyzed by flow cytometry for expression of the various DC markers. For analysis of regulatory T cells, splenocytes were isolated from the recipient mice and subsequently co-stained with CD4, CD25 or CD3, CD8a CD25 antibodies. The cells were further stained with Foxp3 using established techniques [51]. Cells were then analyzed using FACS flow cytometer as previously reported [52].

### Mixed Lymphocyte Reaction, Cell Proliferation and Cytokine Quantification

Splenic T cells (purified using nylon wool columns) isolated from naive or grafted C57BL/6 mice serving as responders (2×10^6^/well) were incubated for 7 days in the presence of Mitomycin C-treated (50 µg/ml, Sigma-Aldrich) DCs used as stimulators derived from naive BALB/c mice (4×10^5^/well, DC: T ratio of 1∶5) in 12-well flat bottom plates (Nunc, Roskilde, Denmark). Cultures were kept at 37°C in 5% CO_2_ atmosphere. Supernatants were recovered after 7 days for determination of IL-10 production. Cytokine level was quantified using Mouse IL-10 ELISA MAX™ Deluxe. T-cell proliferation was assessed by serial dilution of the intracellular CFSE dye as described previously [53].

### Western Blot Analysis

Proteins (50 µg) extracted from cardiac allograft were subjected to electrophoresis on a 12% SDS–PAGE and then transferred onto PVDF membranes. The membranes were then incubated with primary antibodies for Beclin-1, LC3B and GAPDH at 37°C for 2 h, respectively. The blots were visualized using enhanced chemiluminescence (Amersham).

### Immunohistochemistry

After deparaffin and rehydration, the paraffin-embedded heart sections (5 µm) were treated with 3% H_2_O_2_ for 5 min. The non-specific proteins were blocked with 5–10% goat serum for 10 min. For Beclin-1 staining, specimens were incubated with a rabbit anti-mouse Bcelin-1 Monoclonal antibody (1∶200) at 4°C overnight, followed by incubation with a HRP conjugated goat anti-rabbit secondary antibody. The sections were finally incubated with DAB chromogenic substrate and counterstained with hematoxylin.

### Adoptive Transfer Experiments

Total T cells were first enriched with T cells using nylon wool columns, and then subjected to isolation of mouse CD4^+^ T cells using the (L3T4) MicroBeads, and CD8^+^ T cells using the mouse CD8a (Ly-2) MicroBeads as instructed (Miltenyi Biotec, CA)., pDCs were isolated using a mouse Plasmacytoid Dendritic Cell Isolation Kit II as instructed (Miltenyi Biotec, CA). The purified CD4^+^ T cells (5×10^6^), CD8^+^ T cells (5×10^6^), pDCs (5×10^5^) and total splenocytes (5×10^6^) originated from recipients with long-term survival allograft were then adoptively transferred into naive recipients, respectively. One day after the adoptive transfer, the mice were transplanted with cardiac allografts and monitored for allograft survival as described above.

### Statistical Analysis

The data are presented as means ± SD. Statistical differences were determined by one-way ANOVA. Allograft survival differences between groups were determined using the log-rank test. P values less than 0.05 were considered with statistical significant.
